# Case Report: Surgical challenges and insights in a child with a blunt left diaphragmatic and pericardial rupture and heart subluxation

**DOI:** 10.3389/fsurg.2024.1369255

**Published:** 2024-07-17

**Authors:** Melaku Tessema Kassie, Motuma Gonfa Ayana, Ruth Betremariam Abebe, Befikadu Molalign Abebe

**Affiliations:** ^1^ Department of Surgery, College of Medicine and Health Sciences, University of Gondar, Gondar, Ethiopia; ^2^ College of Medicine and Health Sciences, University of Gondar, Gondar, Ethiopia

**Keywords:** blunt trauma, diaphragmatic rupture, pericardial tear, pediatric trauma, heart subluxation

## Abstract

**Introduction:**

Blunt diaphragmatic rupture (BTDR) is a rare condition that can occur in children following high-energy blunt thoracoabdominal trauma. In less than 1% of the cases, pericardial rupture can coexist with a BTDR. A coexistence of BTDR and pericardial rupture can result in displacement of the heart and is associated with high mortality. Clinical presentation is non-specific and requires a high index of suspicion for early management.

**Case presentation:**

A 4-year-old child presented to the emergency unit of our hospital following high-energy trauma with severe respiratory distress. Initially, a left-side chest tube was inserted, but it resulted in no clinical improvement. A chest x-ray showed a collapse of the left lung with a herniation of bowel loops into the left hemithorax. An exploratory laparotomy was done, which revealed a 10 cm × 4 cm defect in the left hemidiaphragm with a medial extension involving the pericardium. The fundus of the stomach and left lobe of the liver were displaced into the pericardial space, pushing the cardiac apex posteriorly to the right side. Concomitantly, the transverse colon and small bowel were displaced into the left pleural space. After the reduction of the herniated abdominal viscera back into the peritoneal cavity, the pericardial sac was repaired by employing an interrupted resorbable suture, while the diaphragmatic defect was repaired by using a horizontal mattress. No other injuries were identified and the abdomen was closed in layers.

**Conclusion:**

BTDR with pericardial rupture is an elusive condition with a high mortality rate that necessitates a high index of clinical suspicion. Early surgical repair of the defect with a reduction of herniated organs can reduce morbidity and mortality.

## Introduction

Diaphragmatic rupture following blunt abdominal trauma is a rare case scenario, with the rate of incidence ranging from 1% to 7% (1). Incidence is particularly low in children, with less than 1% of pediatric trauma resulting in diaphragmatic rupture (2, 3). Since most incidences of blunt traumatic diaphragmatic rupture (BTDR) result from high-energy traumas such as motor vehicle accidents or falls from height, patients often present with severe injuries to the other visceral organs (2, 4–7). Meanwhile, the coexistence of a pericardial rupture is very rare, with the rate of incidence ranging from 0.2% to 3.3% (4). Pericardial ruptures are associated with a high mortality rate (1, 8, 9). Communication between the abdominal cavity and the pericardial space through the tear can allow a herniation of abdominal organs into the chest cavity and pericardial space, resulting in cardiac tamponade, respiratory compromise, or gastrointestinal obstruction (1, 8). The management of choice is early reduction of hernia contents and repair of the defect (1, 4, 10). In this study, we report a rare case of a patient with BTDR with a herniation of the abdominal viscera into the chest cavity and an isolated pericardial rupture with a subluxation of the heart.

## Case report

**Figure 1:**
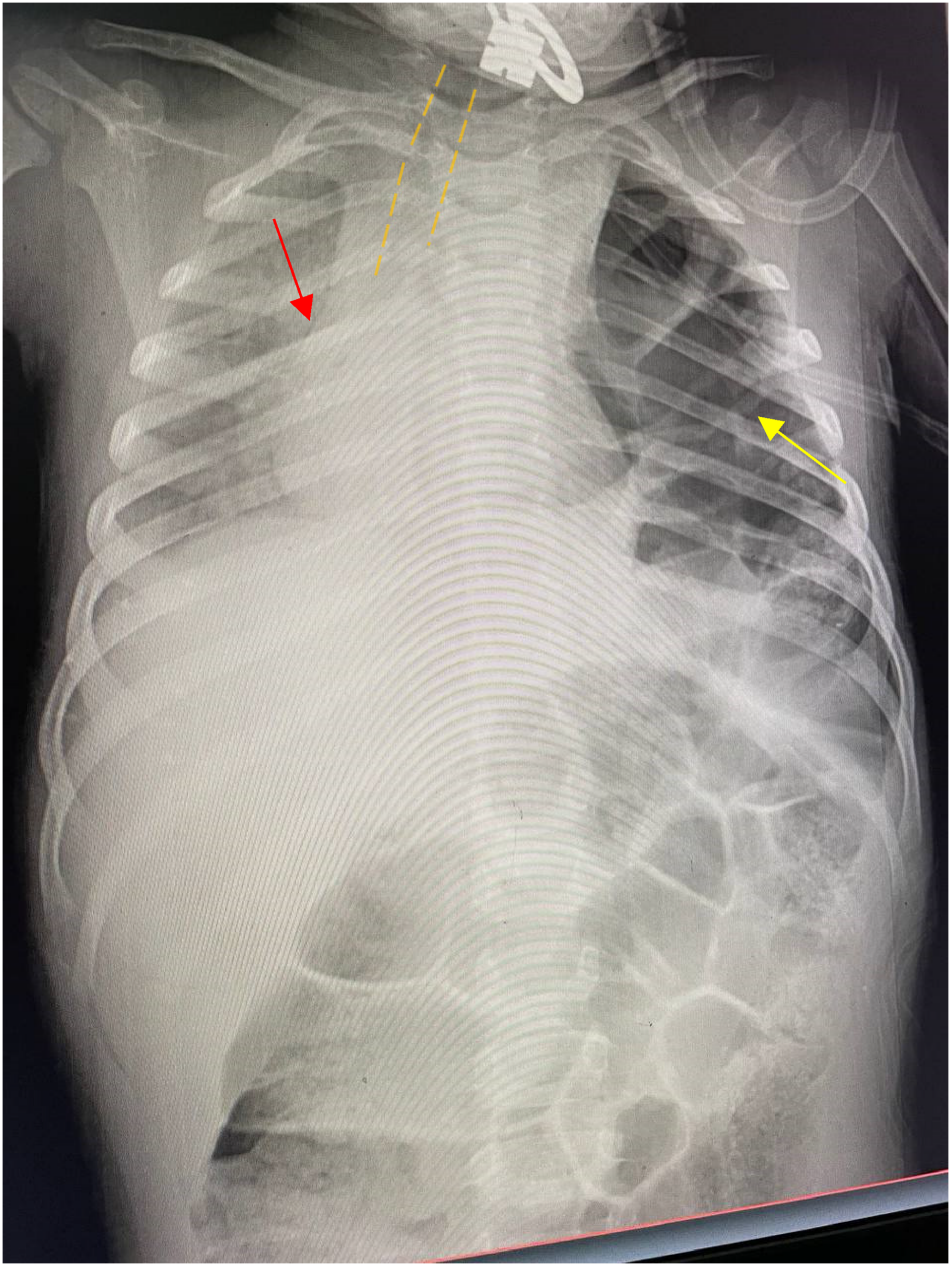
The yellow arrow indicates bowel loops with haustral markings and gas in the left hemithorax. The red arrow indicates a significant shift of the mediastinum to the right side. The image also shows significant tracheal deviation outlined by the yellow dashed line.

A 4-year-old male child presented to the emergency unit of our hospital after he was hit by a high-velocity vehicle. At the emergency unit, he showed signs and symptoms of respiratory distress with RR 66 breaths/min and an SpO2 of 66% at room air and 72% on a 10 L facemask. His blood pressure was 88/40 mmHg and pulse rate was PR 144 beats per minute and regular. The child showed no signs of head injury or a grossly visible fracture. A physical examination revealed absent air entry and hyperresonance on the left side of the chest with abrasion over his left chest wall and flank. The initial impression was a tension pneumothorax, following which a tube thoracostomy was done on the affected side. However, there was no gush of air or blood following tube thoracostomy nor any clinical improvement. Subsequently, a chest x-ray revealed left lung compression, herniation of bowel loops into the hemithorax, and mediastinal shift to the right side (Figure 1).

**Figure 2:**
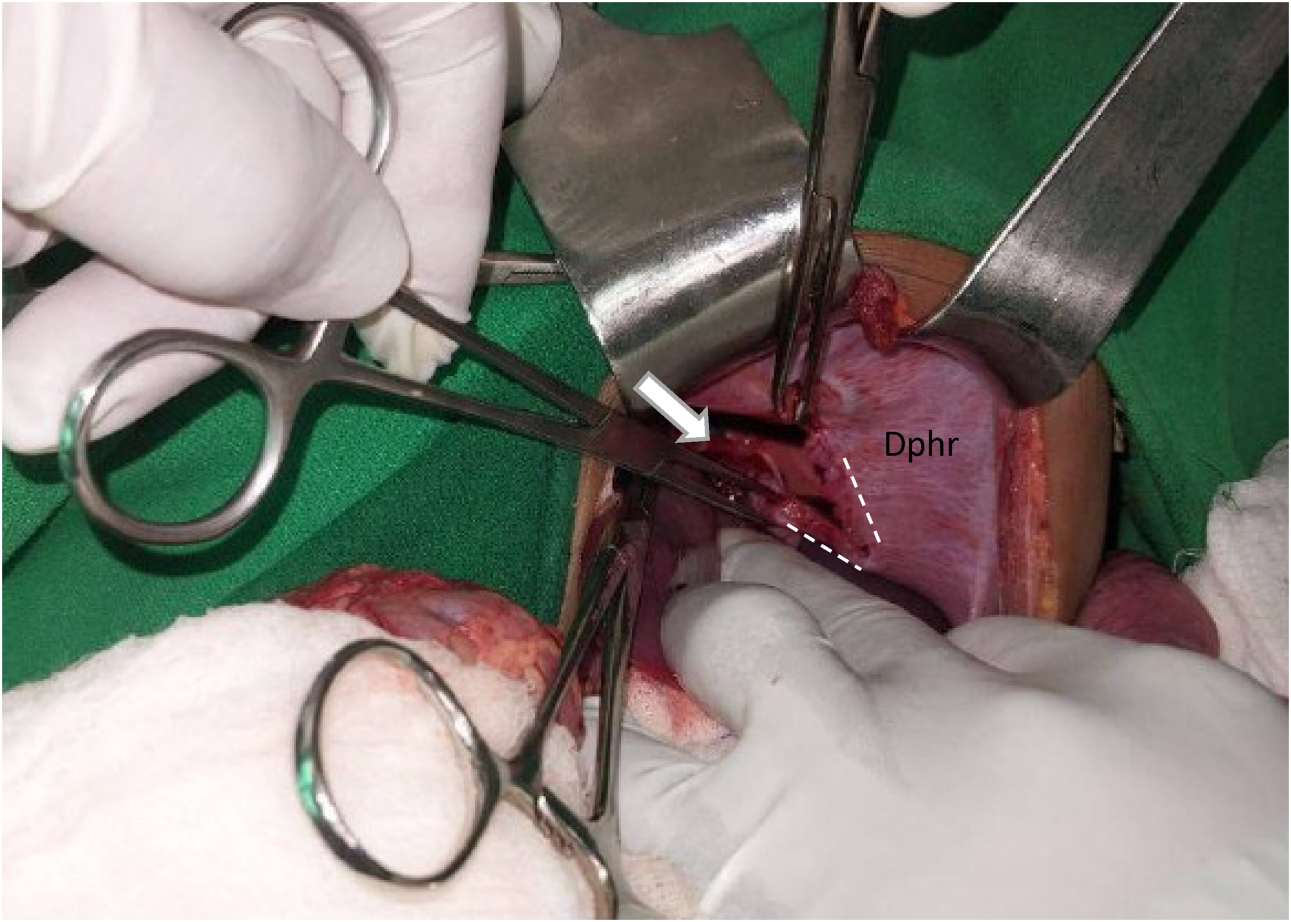
The white arrow indicates the pericardial sac tear. The white dashed line outlines the diaphragmatic defect. “Dphr” labels the left hemidiaphragm.

The patient was transferred to the operating room for an emergency exploration. A left transverse abdominal incision was performed. About 200 ml of blood in the peritoneal cavity with no gross contamination was identified. There was also a 10 cm × 4 cm left hemidiaphragmatic defect with medial extension resulting in a 4 cm × 2 cm pericardial sac defect (Figure 2).

**Figure 3:**
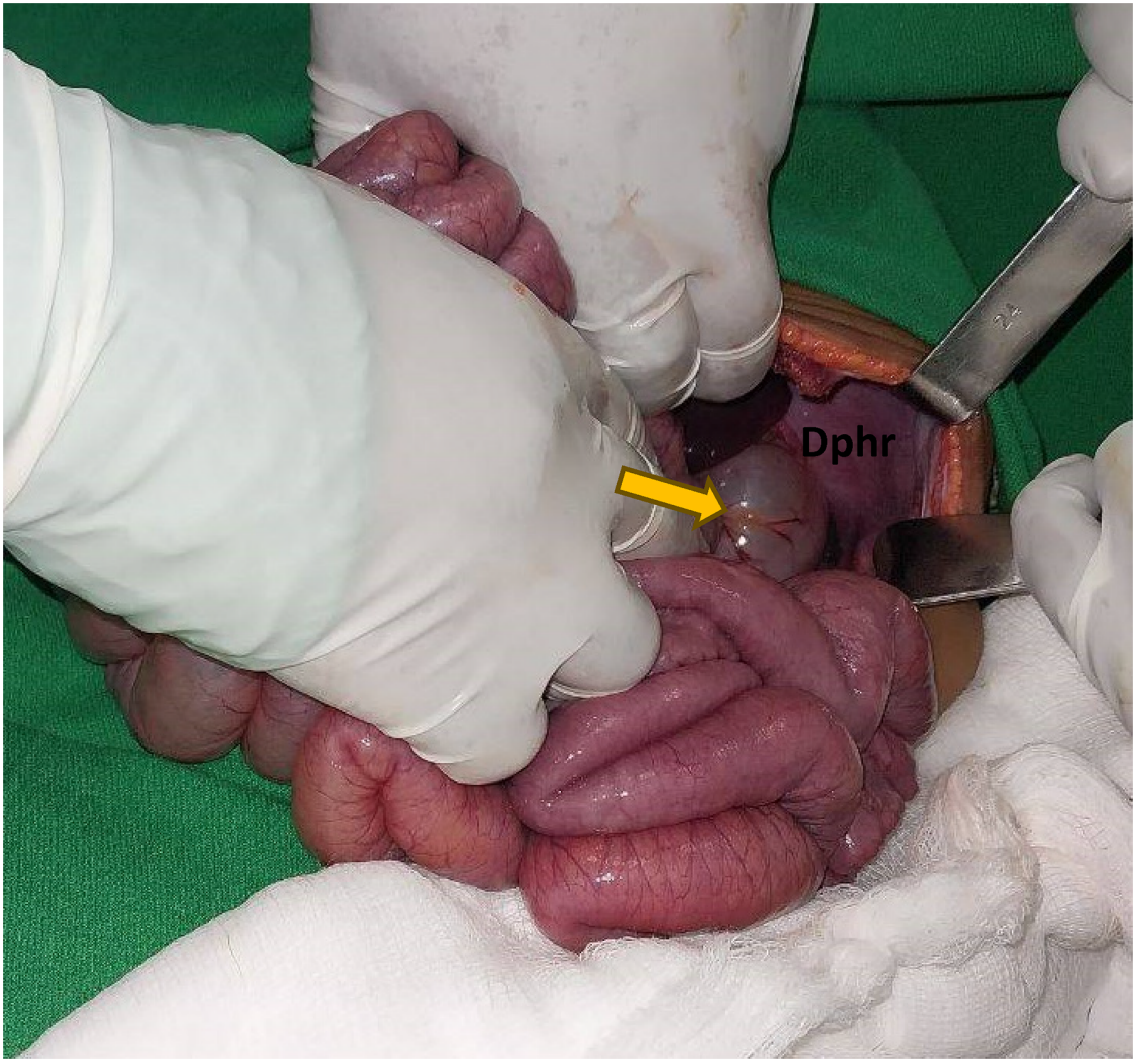
The yellow arrow indicates the transverse colon, and DPHR indicates the diaphragm.

The transverse colon and proximal part of the small bowel herniated into the pleural cavity through the left hemi diaphragmatic defect with a contusion of the transverse colon at the level of the defect (Figure 3). However, there was no bowel perforation.

**Figure 4:**
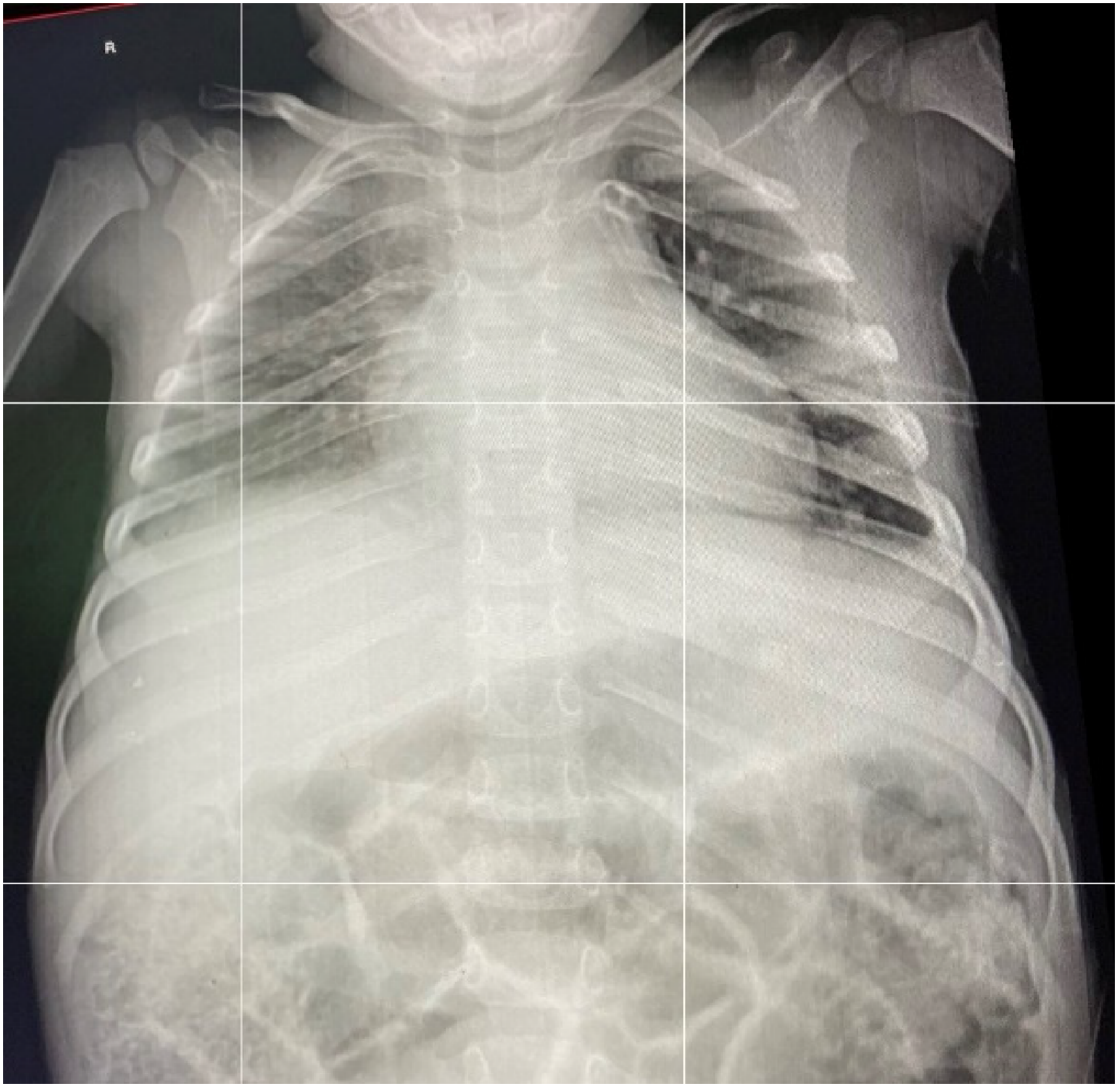
An AP chest x-ray taken 48 h postoperation prior to the removal of the chest tube.

The edge of the left lobe of the liver and fundus of the stomach were uprooted into the pericardial space through the defect in the pericardium, pushing the cardiac apex posteriorly into the right side, but no myocardial contusion was seen. The herniated abdominal viscera were reduced into the peritoneal cavity. Recruitment of the collapsed left lung was done until there was complete expansion. No signs of air leak were evidenced post expansion. The pericardial defect was primarily closed with interrupted resorbable sutures. The diaphragmatic defect was primarily closed with an interrupted horizontal mattress. The integrity of the repair and diaphragmatic motion was then checked via large tidal volume administration. No other visceral injuries were identified, and the abdominal wall was closed in layers. A chest tube was kept in place for 48 h and removed after the evaluation of a control chest x-ray (Figure 4).

## Discussion

A traumatic diaphragmatic rupture is the loss of integrity of the diaphragm due to a sudden increase in intra-abdominal pressure from trauma (10–12). The most common site of rupture is at the junction of the muscle and tendon (3). Traumatic diaphragmatic rupture accounts for less than 0.5% of all trauma cases and 1.9% of all blunt traumas (11). The rate of incidence of BTDR is lower in the pediatric age group, standing at 0.18% of all pediatric traumas (2). Most BTDRs occur in the left posterolateral section of the diaphragm owing to the cushion effect of the liver on the right side (5, 8). A systematic review by Theodorou et al. showed a mortality rate of 2.8% among pediatric patients directly as a result of diaphragmatic rupture and associated herniation (2). The overall mortality rate was reported to be 33% among pediatric patients in studies by the National Trauma Data Bank (NTDB) (2). Patients with BTDR often present with injuries to the abdominal and chest organs (5). One study found that liver and spleen injuries occurred in 40%–60% of pediatric patients with BTDR (2).

Although injury to different organs is commonly associated with BTDR, a pericardial tear is very rare and is associated with a high mortality rate reaching up to 64% (4, 11). An isolated pericardial tear in a patient with BTDR is even rarer (12). However, the patient in this study presented with an isolated pericardial rupture with BTDR. Following blunt trauma, most pericardial ruptures occur in the left pleuropericardial region (12). Pericardial rupture is an elusive diagnosis, with most cases identified intraoperatively (5, 6). Similarly, the defect in the pericardial sac of our patient was identified intraoperatively. Most patients with pericardial rupture are asymptomatic and show signs and symptoms only when there is an associated herniation of the heart or when bleeding occurs (5). Large defects in the pericardium can result in dislocation or torsion of the heart (12). In our patient, there was a subluxation of the cardiac apex on the right side and the defect was closed, with herniated abdominal viscera preventing displacement of the heart out of the pericardial space through the defect.

The most common clinical symptoms of BTDR are signs and symptoms of respiratory distress followed by abdominal pain (2). Signs and symptoms of respiratory compromise can lead to a misdiagnosis of the pneumothorax, resulting in the insertion of a chest tube and iatrogenic injury to the herniated organ (12). In our patient, a similar misdiagnosis was made, and a chest tube was wrongfully inserted. Fortunately, there was no iatrogenic injury to the herniated bowel segments. Plain films of the chest and/or abdomen are the most common imaging methods used in acute cases, with sensitivity ranging from 27% to 73% (5). Findings on plain films associated with pericardial involvement include changes in the cardiac silhouette or axis, signs of pneumopericardium, and pericardial effusion (12). Computed tomography (CT) scans are more specific and sensitive than other imaging modalities to identify chest and abdominal injuries (5).

BTDR should be urgently repaired upon diagnosis, especially in left-sided diaphragmatic ruptures. Operative repair via the abdominal approach is the most common management for primary repair of the diaphragm, although the thoracic approach is recommended in cases of delayed diagnosis due to intra-abdominal adhesions from chronicity (2, 10, 11).

## Conclusions

Diaphragmatic tear following blunt trauma is rare and is often associated with polytrauma. An isolated pericardial involvement is uncommon and difficult to detect before operation. As such, a high level of suspicion is required when evaluating patients with blunt trauma and respiratory distress. Emergent surgery with a reduction of herniated viscera and primary closure of diaphragmatic and pericardial defects can result in better patient outcomes.

## Data Availability

The original contributions presented in the study are included in the article/Supplementary Material, further inquiries can be directed to the corresponding author.
